# STAT3 Contributes To Oncolytic Newcastle Disease Virus-Induced Immunogenic Cell Death in Melanoma Cells

**DOI:** 10.3389/fonc.2019.00436

**Published:** 2019-05-29

**Authors:** Xiaoyan Shao, Xueke Wang, Xianling Guo, Ke Jiang, Tian Ye, Jianhua Chen, Juemin Fang, Linaer Gu, Sitong Wang, Guirong Zhang, Songshu Meng, Qing Xu

**Affiliations:** ^1^Department of Medical Oncology, School of Medicine, Shanghai Tenths People's Hospital, Tongji University, Shanghai, China; ^2^Department of Oncology, Dermatology Hospital, TongJi University, Shanghai, China; ^3^Tongji University Cancer Center, Shanghai, China; ^4^Dalian Medical University Cancer Center, Institute of Cancer Stem Cell, Dalian, China; ^5^Central laboratory, Cancer School of Medicine, Liaoning Cancer Hospital and Institute, Hospital of China Medical University, Shenyang, China

**Keywords:** newcastle disease virus, immunogenic cell death, melanoma, signal transducer and activator of transcription 3, virotherapy

## Abstract

**Background:** Oncolytic viruses (OVs) are emerging as potent inducers of immunogenic cell death (ICD), releasing danger-associated molecular patterns (DAMPs) that induce potent anticancer immunity. Oncolytic Newcastle disease virus (NDV) has been shown to educe ICD in both glioma and lung cancer cells. The objective of this study is to investigate whether oncolytic NDV induces ICD in melanoma cells and how it is regulated.

**Methods:** Various time points were actuated to check the expression and release of ICD markers induced by NDV strain, NDV/FMW in melanoma cell lines. The expression and release of ICD markers induced by oncolytic NDV strain, NDV/FMW, in melanoma cell lines at various time points were determined. Surface-exposed calreticulin (CRT) was inspected by confocal imaging. The supernatants of NDV/FMW infected cells were collected and concentrated for the determination of ATP secretion by ELISA, HMGB1, and HSP70/90 expression by immunoblot (IB) analysis. Pharmacological inhibition of apoptosis, autophagy, necroptosis, ER Stress, and STAT3 (signal transducer and activator of transcription 3) was achieved by treatment with small molecule inhibitors. Melanoma cell lines stably depleted of STAT3 were established with lentiviral constructs. Supernatants from NDV-infected cells were intratumorally injected to mice bearing melanoma cells-derived tumors.

**Results:** Oncolytic NDV induced CRT exposure, the release of HMGB1 and HSP70/90 as well as secretion of ATP in melanoma cells. Inhibition of apoptosis, autophagy, necroptosis or ER stress attenuated NDV/FMW-induced release of HMGB1 and HSP70/90. Moreover, NDV/FMW-induced ICD markers in melanoma cells were also suppressed by either treatment with a STAT3 inhibitor or shRNA-mediated depletion of STAT3. Of translational importance, treatment of mice bearing melanoma cells-derived tumors with supernatants from NDV/FMW-infected cells significantly inhibited tumor growth.

**Conclusions:** Our data authenticate that oncolytic NDV/FMW might be a potent inducer of ICD in melanoma cells, which is amalgamated with several forms of cell death. We also show that STAT3 plays a role in NDV/FMW-induced ICD in melanoma cells. Together, our data highlight oncolytic NDV as propitious for cancer therapeutics by stimulatingan anti-melanoma immune response.

## Introduction

The recent approval of a modified herpes virus (T-Vec) for the treatment of advanced melanoma patients points to the potential of oncolytic viruses (OVs)-mediated therapy of cancers (1–4). Notably, oncolytic virotherapy improves immune checkpoint blockade-based immunotherapy in several types of cancers as demonstrated in a few preclinical and clinical trials (5–7). A potential mechanism for the enhanced clinical benefit of immune checkpoint blockade by this new combinatorial strategy, i.e., OVs combination with immune checkpoint inhibitors, is that OVs can recondition the tumor microenvironment (8–11). Particularly, in addition to their direct cytolytic effects (a mechanism known as oncolysis), a growing number of OVs are now being acknowledged for their capacity to induce immunogenic cell death (ICD) of cancer and stromal cells and to release tumor-associated antigens (12–19), which re-educates the host's immune system to induce antitumor immunities (8, 20–22). ICD is characterized by the secretion, release, or surface exposure of damage-associated molecular patterns (DAMPs), OV-derived pathogen-associated molecular pattern (PAMP) molecules and inflammatory cytokines (23–26). The well-characterized DAMPs as hallmarks of ICD generally include surface-exposed endoplasmic reticulum (ER) chaperone calreticulin (ecto-CRT), secretion of ATP and release of high mobility group box 1 (HMGB1) (27–30). Other DAMPs such as heat-shock proteins (HSP90 and HSP70) and ER sessile proteins are also exposed on the outer membrane of the dying cells or released (31–33). After secretion, these DAMP molecules bind to their receptors CD91 (CRT), P2RX7 (ATP), and TLR4 (HMGB1) on dendritic cell, which support their recruitment and improve their antigen uptake and capacity to stimulate the T cells (34–36). Given the emerging role of ICD in OVs-mediated immunotherapy, it is increasingly important to fully understand how OVs trigger ICD in infected cancer cells, thereby maximizing ICD-triggered antitumor immunity.

Among the OVs that have completed the first phase I/II clinical trials, Newcastle disease virus (NDV), which is an avian paramyxovirus, has achieved longstanding benefit as an oncolytic agent in patients of advanced cancers (37–41). Recent studies by Zamarin et al. showed that intratumoral therapy with NDV sensitizes the tumors to the efficacy of CTLA-4, PD-1, and PD-L1 blockade in animal models (42, 43). In addition, oncolytic NDV has been shown to trigger tumor-specific immune memory in orthotopic glioma through the induction of ICD (44). Our lab recently reported that an oncolytic NDV strain FMW (here as NDV/FMW) is a potent ICD-inducer in lung cancer cells (45, 46). However, whether oncolytic NDV induces ICD in other cancer types and the underlying mechanism (s) remain to be explored.

In the present study, our aim was to explore whether NDV/FMW induces ICD in melanoma cells and its regulation. We show that NDV/FMW triggers ICD in both cultural melanoma cells and in mouse models, which can be modulated by targeting signal transducer and activator of transcription 3 (STAT3).

## Materials and Methods

### Cell Lines and Virus

The human melanoma cell lines A375 and C8161 as well as chicken embryo fibroblast cell line DF1 and human embryonic kidney cells (293T) were originally obtained from American Type Culture Collection (ATCC) and kept in our lab. These cells were cultured at 37°C and 5% CO_2_ in DMEM, supplemented with 10% fetal bovine serum (FBS). Oncolytic NDV strain, NDV/FMW, which has been previously shown to induce cytotoxic effects in A549/DDP and parental cells (46), was used throughout the study. Virus titer was expressed as log_10_ of 50% the infective dose (TCID_50_) in culture.

### Antibodies and Regents

Anti-calreticulin (CRT) and anti-p62/SQSTM1 antibodies were purchased from Abcam. Anti-HN, anti-HSP90, and anti-STAT3 antibodies were obtained from Santa Cruz. Anti-β-actin and goat anti-rabbit antibody were purchased from Proteintech. Goat anti-mouse and Goat anti-rabbit antibodies for immunoblot analysis were obtained from Bioworld. The secondary antibodies of Alexa 488, Alexa 568 and Alexa 647 for immunofluorescence were obtained from Invitrogen. The following antibodies from Cell Signaling Technology were used: HMGB1, HSP70, poly (ADP-ribose) polymerase (PARP), p-eIF2α, p-STAT3 (Y705), Bcl-xl, Mcl-1, β-catenin. Mitoxantrone (MTX), Necrostain-1 (Nec-1), Z-VAD-FMK (Z-VAD), chloroquine (CQ), GSK2606414 (GSK), and C188-9 were obtained from Selleckchem. Recombinant interleukin-6 (IL-6) were obtained from PeproTech. Drugs were dissolved in dimethyl sulfoxide (DMSO) as stock solutions and stored at −20°C. ENLITEN®ATP Assay System Bioluminescence Detection Kit for ATP Measurement (#FF2000) was purchased from Promega. PI, Pierce®Protein Concentrator 2–6 mL/10K filters were purchased from Thermo Scientific. HMGB1 ELISA Kit II (#L534) was purchased from SHINO-TEST CORPORATION. Trypan blue dye was obtained from Sigma.

### Virus Infection

Melanoma cell lines were infected with NDV/FMW at a multiplicity of infection (MOI) of 1, or mock-infected with PBS, at 37°C in serum-free DMEM for 1 h. The cells were washed three times with PBS and incubated at 37°C in DMEM supplemented with 1% FBS. For pharmacological modulation of STAT3 signal pathway, cells were treated with C188-9 (0.9 μM) and IL-6 (30 ng/mL) for 1 h prior to virus infection. Subsequently, the cells were infected with NDV/FMW in the presence or absence of various compounds for 1 h and then cultured in fresh DMEM containing C188-9 or IL-6 for the indicated times. For experiments that involved the determination of virus yield, tumor cells were infected with NDV/FMW at an MOI of 0.01, and multi-step viral growth curves were measured as previously described (47).

### Lentiviral Constructs and Stable Cell Lines

A375 and C8161 cell lines stably depleted of STAT3 were established with the lentiviral construct. The 293T cells were transfected with packaging plasmid (PSPAX and PMD2G) and knockdown plasmid (STAT3 shRNA) or control plasmid (non-coding shRNA) for 48 h, the supernatants were collected and infected A375 and C8161 cells for 48 h. Stable clones were then selected using puromycin (Sigma). The selected cell populations were subjected to immunoblotting to investigate the silencing efficiency. The plasmids of STAT3 shRNA (#6774) and non-coding shRNA (RHS4346) were obtained from CCSB-Broad Lentiviral Expression Library (Dharmacon).

### Preparation of Concentrated Supernatants

The supernatants (6 mL) of NDV/FMW infected and uninfected cells were collected and placed into the PierceProtein Concentrator 2–6 mL/10 K filters (ThermoFisher Scientific), and then the samples were centrifugated at 1,000 rpm until the volume from 6 mL to 100 μl. The concentrated cell-free supernatants were subjected to immunoblot (IB) analysis to examine secreted HMGB1 and HSP70/90.

### Immunoblot Analysis

A375 and C8161 cells were infected or uninfected with NDV/FMW at MOI of 1 for 12, 24 and 48 h, and then cells were placed on ice, washed with cold PBS, harvested using a scraper and lysed in lysis buffer for 25 min. Cell lysates were centrifuged at 12,000 g for 10 min at 4°C and supernatants were subjected to western blot analysis. Cell lysates and the concentrated cell-free supernatants were loaded on SDS-PAGE and transferred onto a nitrocellulose membrane. The membranes were blocked with 5% non-fat milk in TBST (Tris-buffered saline tween) at room temperature for 3 h and incubated with primary antibodies at 4°C overnight. Following washing three times with TBST, the membranes were incubated at room temperature for 1 h with corresponding horseradish peroxidase-conjugated secondary antibodies. The blots were detected using an ECL Western Blot Substrate kit according to the manufacturer's protocol.

### Trypan Blue Exclusion Assay

A375 and C8161 cells were infected and uninfected with NDV/FMW (MOI = 1) for 48 h, then the cell were collected and diluted in Trypan blue dye by preparing a 1:1 dilution of the cell suspension using a 0.4% Trypan blue solution for 2 min at room temperature. Cell viability was analyzed using hemocytometer. The ratio of unstained cell numbers to total cell numbers was reported as the viability percentage for each cell category.

### Flow Cytometric Analysis

A375 and C8161 cells were infected or uninfected with NDV/FMW (MOI = 1) for 48 h, and then the cells were harvested with 0.25% trypsin without EDTA, washed twice with ice-cold PBS and resuspended in 500 μL PBS. Subsequently, cells were incubated with an anti-CRT antibody for 2 h on ice, following by washing and incubated with AlexaFluor 488-conjugates for 30 min, then PI was added into cells with the final concentration of 1 μg/mL and the cells were incubated for 15 min in dark. The samples were analyzed by flow cytometry analysis. Surface expression of CRT was analyzed by flow cytometry.

### Determination of ATP Secretion

ATP secretion was measured with the ENLITEN ATP Assay (Promega, FF2000) according to the manufacturer's protocol, using a multifunctional enzyme labeling instrument (Enspire2300, Perkin Elmer, USA). The supernatants of A375 and C8161 cells were collected, dying floating cells were removed by centrifugation at 1,000 rpm for 5 min. The 2% bleach (sodium hypochlorite) solution was used to remove bacteria and traces of ATP from the reagent injector and tubing. One hundred microliter of reagent and 100 μl of supernatant both added to 96 wells plate for each assay. According to the ATP standard curve, the ATP concentration of the supernatants was obtained.

### HMGB1 and Enzyme-Linked Immunosorbent Assays

The HMGB1 ELISA Kit was used to detect HMGB1 release. After drawing a standard curve series using sample diluents and standard, the supernatants of cells were collected and centrifugated to remove floating cells. One hundred microliter of sample diluents were added to each well, 10 μL of sample diluent to zero well for the negative control, and 10 μL of samples were added to each well. After shaking the plate with a plate mixer, all wells were covered with a plate seal and incubated for 24 h at 37°C. All wells were washed 5 times with wash solution (400 μL/well). After the final wash, the plates were turned over and gently tap 5 times on a lint-free paper towel to remove any remaining wash buffer. Then 100 μL of POD-conjugate solution was added to each well and incubated for 2 h at 25°C. After washing, 100 μL of substrate solution was added to each well and incubated for 30 min at room temperature. Then 100 μL of stop solution was added to each well in the same sequence, and the absorbance of each well at 450 nm was tested by a luminometer.

### Confocal Imaging

For immunofluorescence microscopy, cells were placed on coverslips (NEST, 801008). Virus-infected and uninfected cells were washed twice with ice-cold PBS and fixed in 4% paraformaldehyde for 10 min and incubated at room temperature for 60 min in 3% Bovine Serum Albumin (BSA). The cells were incubated with primary antibody overnight at 4°C. Following washing three times with PBS, the cells were incubated with secondary antibody in PBS containing 3% BSA at room temperature for 1 h with secondary antibodies. Nuclei were stained with DAPI (5 μg/mL) in PBS. Using a confocal laser microscope (Leica TCS SP5) with a × 60 oil objective to obtain Images. Images from each experiment were acquired using the same exposure time during the same imaging session. The slides were analyzed using the open source Image J (64 Bit for Windows) imaging platform.

### Animal Experiments

BALB/c nude mice (female, 6 weeks old) were obtained from Beijing Vital River Laboratory Animal Technology Co., Ltd. A375 and C8161 cells were subcutaneously inoculated into the flank to induce tumor formation. When tumors reached 100 mm^3^ (after 30 days), tumor-bearing mice were intratumorally inoculated with NDV/FMW. Mice were randomly divided into three groups and five mice were included in each treatment group: (a) vehicle treatment, (b) intratumoral injection with concentrated cell-free supernatants from NDV/FMW-infected cells (the supernatants were ultraviolet-irradiated for 60 min at intensity of 0.15 mW cm^−2^) every 3 days, (c) intratumoral injection with NDV/FMW (1 × 10^7^ TCID_50_ per dose) every 3 days. Tumor growth and survival were measured every 5 days by digital calipers. Tumor volume was calculated as [(greatest diameter) × (smallest diameter)^2^/2]. The experiments were scheduled to last for 90 days from the day of tumor implant. Any animal whose weight decreased more than 30% from the weight on the first day of treatment, ulcerated or sloughed off, or was moribund was euthanized prior to study termination. At the bottom line of the study, the surviving mice were sacrificed under anesthesia. All procedures involving animals and their care complied with the China National Institutes of Healthy Guidelines for the Care and Use of Laboratory Animals. Ethical approval for the study was granted by the Ethics Committee of Dalian Medical University.

### Statistical Analysis

Statistical analyses were performed using the Student's *t*-test with Microsoft Excel (Microsoft, Redmond, WA, USA). Results were expressed as the Mean ± SD of at least three independent experiments and *p* < 0.05 were considered as statistically significant.

## Results

### Oncolytic NDV Induces CRT Exposure, Release of HMGB1 and HSP70/90 as Well as Secretion of ATP in Melanoma Cells

To explore whether NDV/FMW could elicit ICD in melanoma cells, we first examine whether NDV/FMW could replicate and trigger cell death in melanoma cells. In line with our previous work in lung and thyroid cancer cells (46, 48), NDV/FMW robustly replicated in human melanoma A375 and C8161 cells as evidenced by elevated virus titers and the expression of NDV hemagglutinin-neuraminidase protein (HN) (Supplementary Figures 1A,B). We also observed growth inhibition of NDV/FMW-infected melanoma cells, which was accompanied by cleaved poly (ADP-ribose) polymerase (PARP, apoptosis marker), reduced p62 (autophagy flux indicator) and increased phosphorylation of eIF2α (ER stress marker) (Supplementary Figure 1B and data not shown), indicating that multiple modes of cell death might be involved in NDV/FMW-mediated growth inhibition of melanoma cells.

**Figure 1:**
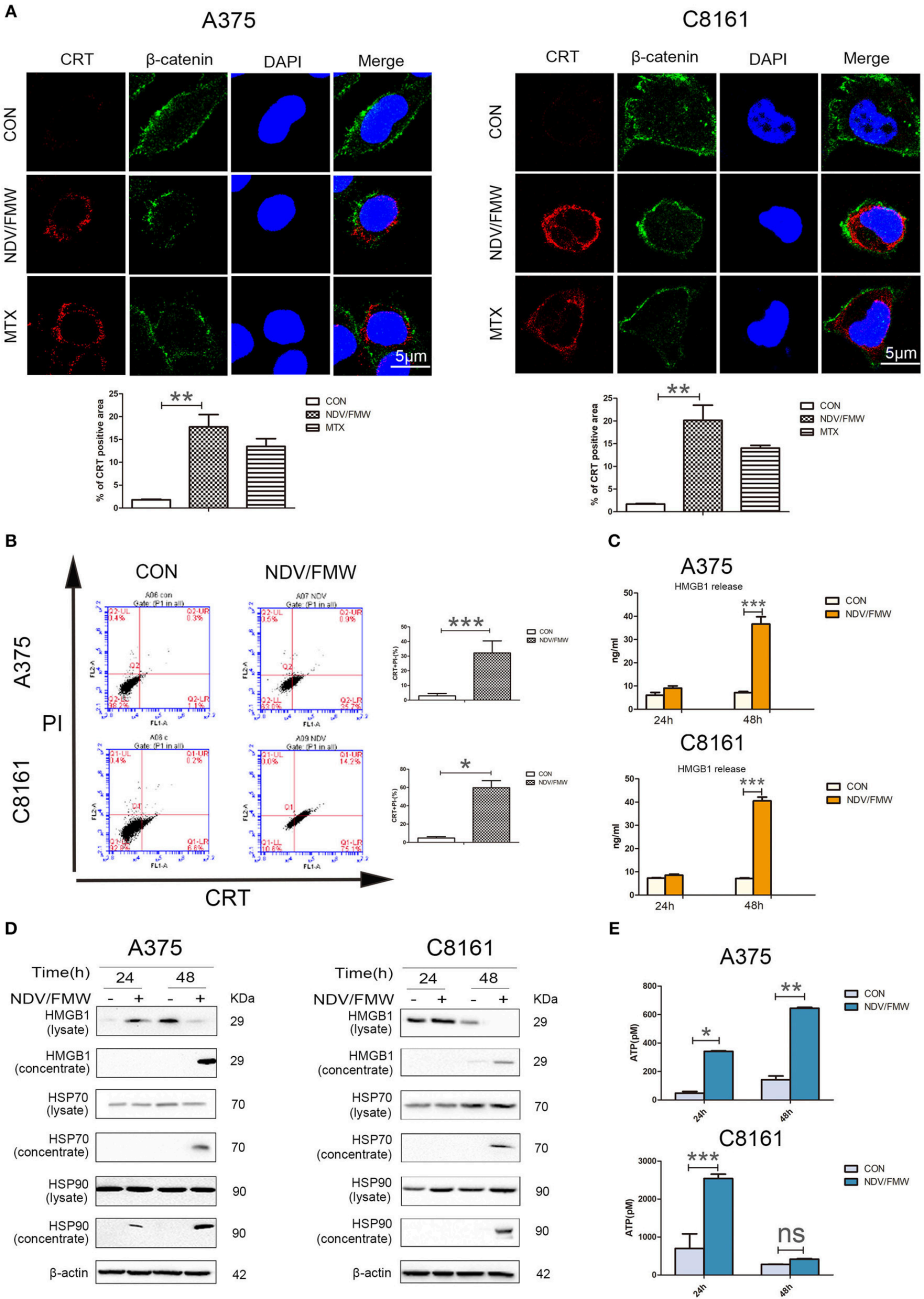
NDV/FMW induces immunogenic cell death in melanoma cancer cells. **(A)** A375 and C8161 cells were infected with or without NDV/FMW (MOI = 1) for 48 h, to assess the translocation of calreticulin (CRT), A375 and C8161 cells were stained with an anti-CRT antibody (Red) and anti-β-catenin antibody (Green), and assessed by confocal imaging at 48 hpi of NDV/FMW (MOI = 1). β-catenin was used as a membrane marker. Mitoxantrine (MTX) was used as a positive control. DAPI was used for nuclear staining (blue). ImageJ software was used to calculate the percentage of CRT positive area (**p < 0.01). Imaging data has been quantified. Images are representative of three independent experiments. **(B)** A375 and C8161 cells were infected as the same in **(A)**, the expression of CRT on the cell membrane were analyzed by flow cytometry to detect CRT in viable, PI-negative cells (*p < 0.05, ***p < 0.001). Representative dot plots (left panel) and quantification data (right panel) are shown. Data are shown for three independent replicates. **(C)** A375 and C8161 cells were infected with or without NDV/FMW (MOI = 1) for 24 and 48 h, release of HMGB1 in NDV/FMW-infected or mock-infected cell supernatants were detected by enzyme-linked immunosorbent (ELISA) (***p < 0.001). Data shown are representative of three independent experiments. **(D)** A375 and C8161 cells were infected as the same in **(C)**, then cell lysates and the concentrated cell-free supernatants were collected. HMGB1 and HSP70/90 expression were measured by immunoblot (IB) analysis. β-actin was used as a loading control. **(E)** A375 and C8161 cells were infected as the same in **(C)**, extracellular ATP was determined by ELISA (*p < 0.05). Data shown are representative of three independent experiments (n.s, not significant).

Given that oncolytic NDV triggered ICD in glioma and lung cancer cells as demonstrated by our previous work and others (44–46), we hypothesized that NDV/FMW would induce ICD in melanoma cells. To test this hypothesis, we measured the ICD markers ATP, HMGB1, and HSP70/90 in supernatants after viral infection and checked the cell surface of infected melanoma cells for CRT expression (ecto-CRT). Treatment with mitoxantrine (MTX) was chosen as a positive control, because MTX was previously described as a legitimate ICD inducer (49). As shown in Figure 1A, confocal imaging of NDV/FMW-infected A375 and C8161 cells revealed an increased exposure of CRT (red) on the cell surface at 24 and 48 post infection (hpi) compared to mock-infected cells. As expected, MTX treatment induced strong exposure of CRT in both melanoma cell lines. We also observed that the NDV envelope protein, HN, was evidently stained with an anti-HN antibody in NDV/FMW-infected cells but not in mock-infected or MTX-treated cells (Supplementary Figure 1C). In addition, NDV/FMW infection-induced CRT exposure in A375 and C8161 cells were further confirmed by flow cytometry analysis (Figure 1B). To detect the secreted DAMPs in NDV/FMW-infected melanoma cells, the cell culture media was collected and concentrated at 24 and 48 hpi. Both ATP secretion and HMGB1 release were determined by ELISA while other released DAMPs were assayed by immunoblotting. As illustrated in Figures 1C,E, NDV/FMW infection of both A375 and C8161 cell lines at 24 or 48 h resulted in an increase of extracellular ATP and HMGB1, respectively, as determined by ELISA assay. In addition, dramatically increased protein levels of both HMGB1 and HSP70/90 were detected in concentrated supernatants of A375 and C8161 cell lines infected with NDV/FMW at 48 hpi (Figure 1D).

### Pharmacological Inhibition of Apoptosis, Autophagy, Necroptosis, and ER Stress Suppresses NDV/FMW-Induced Release of HMGB1 and HSP70/90 in Melanoma Cells

**Figure 2:**
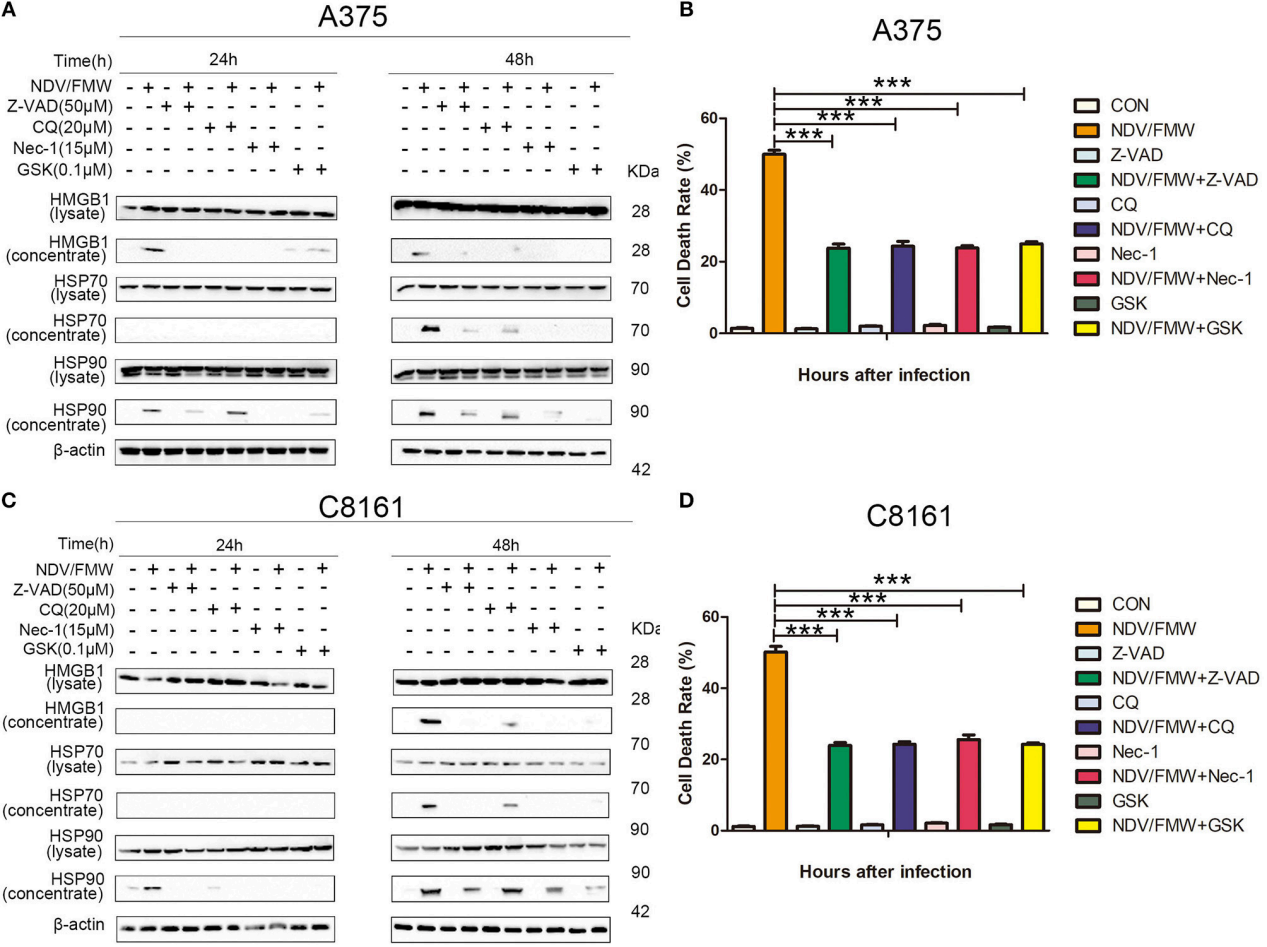
Pharmacological inhibition of apoptosis, autophagy, necroptosis, and ER stress suppresses NDV/FMW-induced immunogenic cell death in melanoma cells. **(A,C)** A375 and C8161 cells were pre-treated with either Z-VAD-FMK (Z-VAD, 100μM), chloroquine(CQ, 20 μM), Necrostain-1 (15 μM), GSK2606414 (GSK, 0.1 μM) or mock-treated for 1 h, following infection of NDV/FMW for 24 and 48 h, then cell lysates and cell-free supernatants (concentrated) were collected. HMGB1 and HSP70/90 were measured by IB analysis. β-actin was used as a loading control. **(B,D)** A375 and C8161 cells were infected as the same in **(A,C)**. The cell death rate of A375 and C8161 cells were obtained by trypan blue staining. Data shown are representative of three independent experiments (***p < 0.001).

The incidence of ICD is generally acknowledged to be tightly connected with apoptosis, autophagy, necroptosis or ER stress (24, 50–54). Our previous study showed that autophagy but not apoptosis or necroptosis contributes to NDV-mediated induction of ICD in lung cancer cells (45). To test whether apoptosis, autophagy, necroptosis or ER stress would play a role in NDV/FMW-triggered ICD in melanoma cells, we pretreated the cells with the pan-caspase inhibitor Z-VAD-FMK (Z-VAD), the autophagy inhibitor chloroquine (CQ), the necroptosis inhibitor Necrostain-1 (Nec-1), and ER stress inhibitor GSK2606414 (GSK), respectively. The effective concentrations of these inhibitors were selected by a dose–response assay for each compound to prevent cytotoxicity (data not shown). Figure 2A shows that the tested four inhibitors all effectively blunted the release of HMGB1 and HSP70/90 in A375 cells exposure to NDV/FMW compared to cells treated with virus alone. Similar results were obtained in C8161 cells treated as in A375 cells (Figure 2C). In addition, to assess the effect of apoptosis, autophagy, necroptosis or ER stress inhibitors on NDV/FMW-induced cell death, Figures 2B,D showing cell death rate by trypan blue assay (Figures 2B,D).

### Pharmacological Inhibition of STAT3 Attenuates NDV/FMW Replication and Oncolytic Cell Death in Melanoma Cells

**Figure 3:**
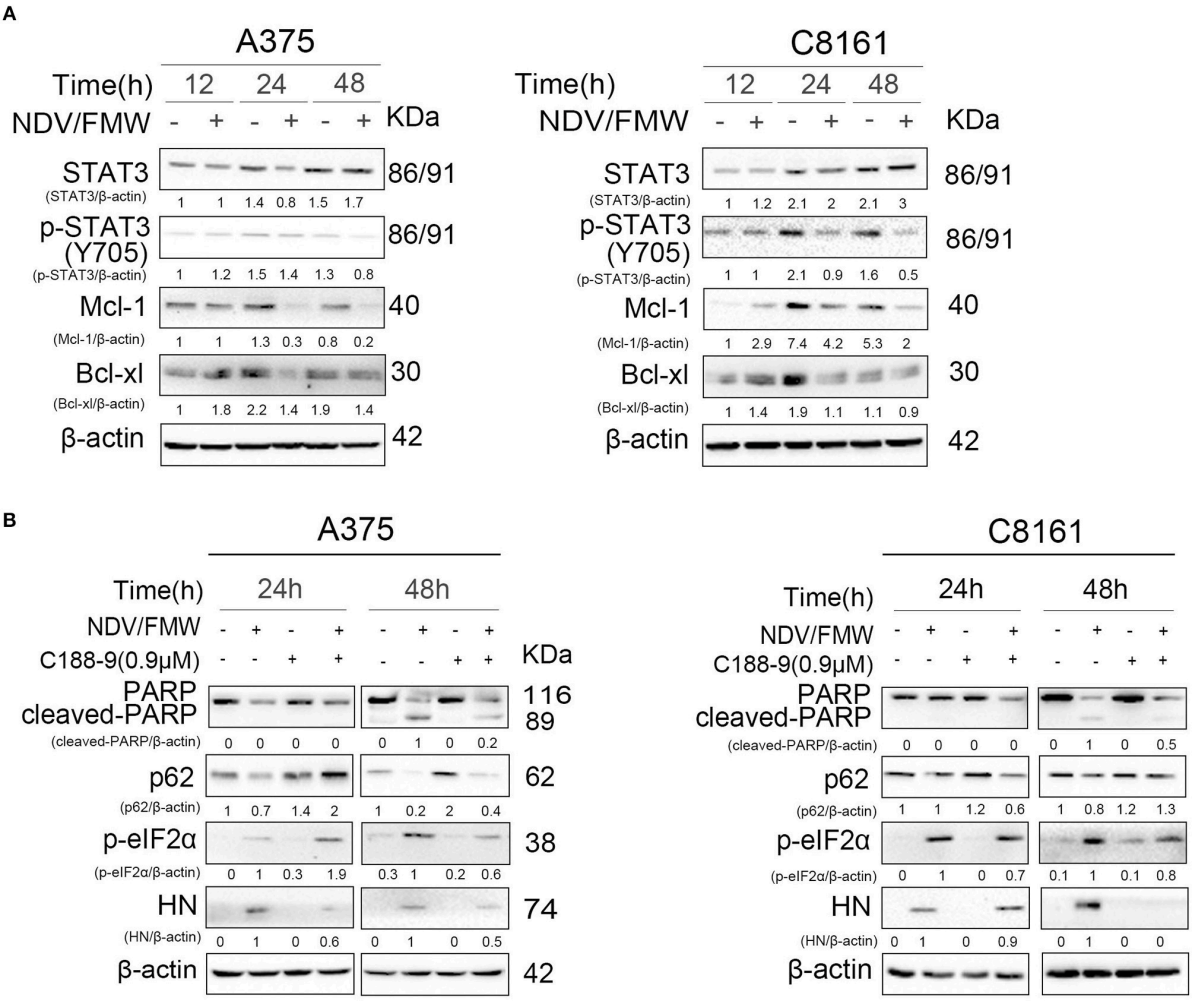
Effects of pharmacological modulation of STAT3 on NDV/FMW-induced apoptosis. **(A)** A375 and C8161 cells were infected with or without NDV/FMW (MOI = 1) for 12, 24, and 48 h. The relative expression of p-STAT3 (Y705), STAT3, Mcl-1, and Bcl-xl was measured by IB analysis. β-actin was used as a loading control. The relative quantity of protein was assessed by Image Lab software. Data shown are representative of three independent experiments. **(B)** A375 and C8161 cells were pre-treated with C188-9 (STAT3 inhibitor) or mock-treated for 1 h, following infection with or without NDV/FMW for 24 and 48 h. The relative expression of cleaved-PARP, p62, p-eIF2α, and HN was determined by IB analysis. β-actin was used as a loading control. The relative quantity of protein was assessed by Image Lab software. Data shown are representative of three independent experiments.

The JAK-STAT3 signaling plays a key role in anticancer immunotherapy and in cytokines such as interleukin-6 (IL-6)-mediated effects in cancer progression (55–58). Our previous work showed that targeting STAT3 can inhibit tumor VEGF expression and angiogenesis in melanoma cells (59). A recent study showed that deletion of STAT3 stimulates one of the hallmarks of ICD in fibrosarcoma cells (58, 60). We thus hypothesized that STAT3 might play a role in NDV/FMW-induced ICD in melanoma cells. To test this, we first examinedthe activation of STAT3 (tyrosine phosphorylation of STAT3 at Y705) in melanoma cells in response to NDV/FMW infection. As shown in Figure 3A, NDV/FMW infection of A375 and C8161 cells resulted in a pronounced decrease in pSTAT3 (Y705) at 48 hpi. Consistently, Mcl-1 and Bcl-xl, two known STAT3 target genes, were downregulated in the infected cells (Figure 3A). We next investigated the effects of STAT3 inhibition on NDV/FMW replication and cell death in melanoma cells. To achieve this, C188-9, a specific STAT3 inhibitor which has been evaluated in early phase clinical trials for advanced-stage cancers (NCT03195699) (61–63), was used through our experiments and its efficacy was validated by the reduction of phosphorylated levels of STAT3 (data not shown). We observed that pretreatment with C188-9 obviously antagonized NDV/FMW infection-induced PARP cleavage, p62 reduction and eIF2α phosphorylation in A375 and C8161 cells at 48 hpi (Figure 3B). Notably, C188-9 treatment reduced the protein levels of NDV protein HN in infected melanoma cells (Figure 3B), indicating that STAT3 inhibition might decrease NDV/FMW replication in melanoma cells.

### STAT3 Inhibition Suppresses NDV/FMW-Induced ICD Markers in Melanoma Cells

**Figure 4:**
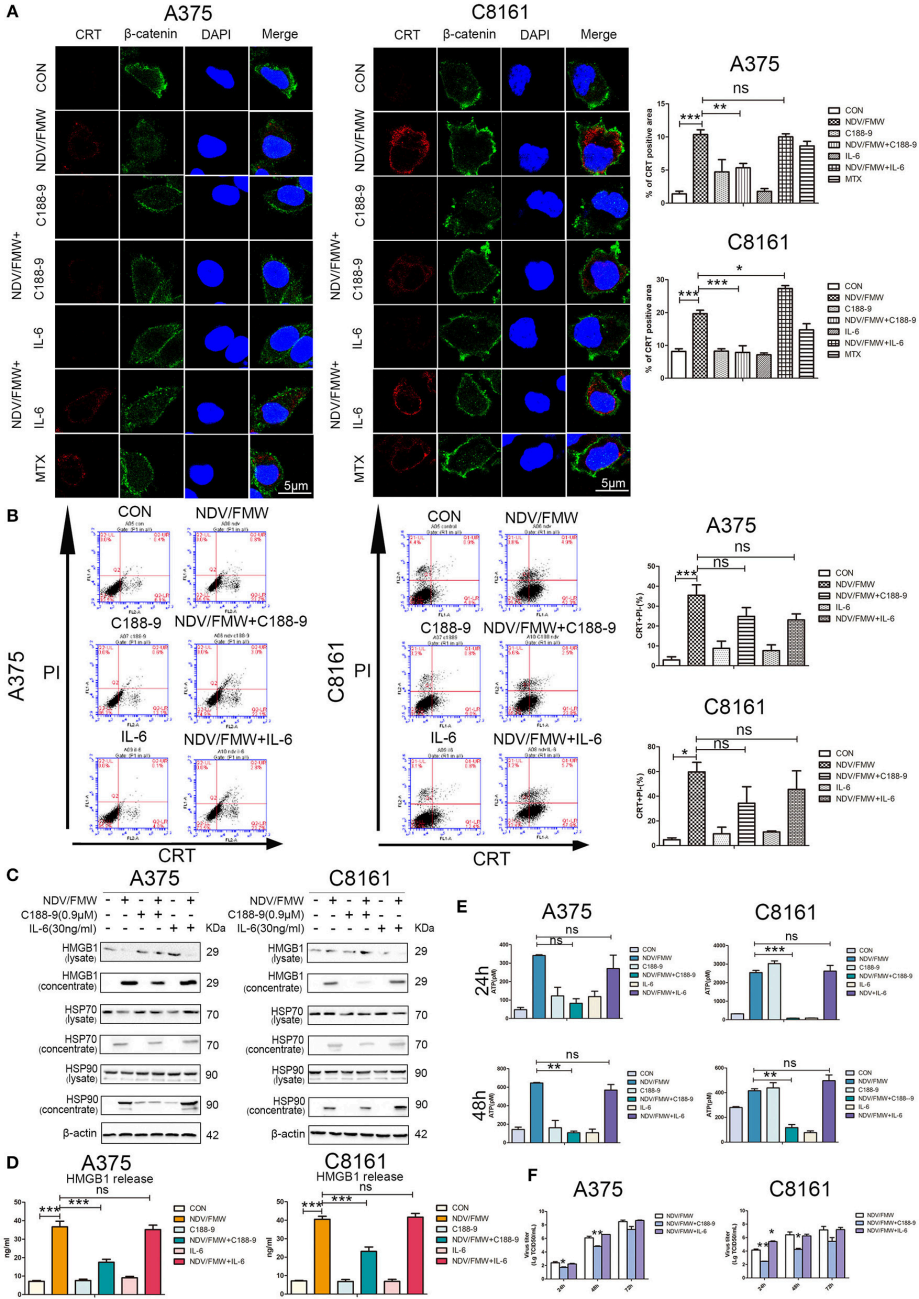
STAT3 inhibition exerts effects on NDV/FMW-induced immunogenic cell death. **(A–E)** A375 and C8161 cells were pre-treated with C188-9 (0.9 μM) and IL-6 (30 ng/mL) and then cells were infected or mock-infected with NDV/FMW (MOI = 1) for 48 h. **(A)** Translocation of CRT (red) was assessed by immunofluorescence staining. β-catenin was used as a membrane marker (green). MTX was used as a positive control. DAPI was used for nuclear staining (blue). Images were obtained using confocal microscopy. ImageJ software was used to calculate the percentage of CRT positive area (**p < 0.01). Images are representative of three independent experiments. **(B)** The cells were stained for the detection of CRT in viable, PI-negative cells by flow cytometry. Quantification data are shown for three independent experimental replicates (*p < 0.05, ***p < 0.001, n.s = not significant). **(C)** Cell lysates and cell-free supernatants (concentrated) were collected for IB analysis. β-actin was used as a loading control. Images are representative of three independent experiments. **(D)** Extracellular HMGB1 was assessed by ELISA in the culture supernatants (***p < 0.001, n.s = not significant). **(E)** Extracellular ATP was measured by ELISA in the culture supernatants (**p < 0.05, ***p < 0.01, n.s = not significant). Data shown are representative of three independent experiments. **(F)** A375 and C8161 cells were pre-treated with C188-9 (0.9 μM) and IL-6 (30 ng/mL) and subsequently infected with NDV/FMW (MOI = 0.01), virus yield was examined at the indicated times. Representative images are shown for three independent experiments. Data are presented as the mean±SD for triplicate assays (*p < 0.05, **p < 0.01).

Having shown that pharmacological inhibition of STAT3 affects NDV/FMW-mediated cell death in melanoma cells, we asked whether STAT3 inhibition could exert effects on NDV/FMW-induced ICD markers. As depicted in Figures 4A,B, and Supplementary Figure 2A pretreatment with C188-9 reduced NDV/FMW infection-elicited CRT exposure, in both A375 and C8161 cells as determined by confocal imaging and flow cytometry, respectively. In addition, exposure to C188-9 decreased ATP secretion and release of HMGB1, HSP70, and HSP90 in A375 and C8161 cells upon NDV infection as assayed by immunoblotting and ELISA respectively, (Figures 4C–E). Of interest, pretreatment with IL-6 enhanced the release of HSP90 in NDV/FMW-treated A375 cells while in NDV/FMW-infected C8161 cells (Figure 4C). In addition, C188-9 treatment decreased virus yield in NDV/FMW-infected cells compared to virus infection alone whereas IL-6 treatment increased virus titers in A375 cells at 48 hpi and in C8161 cells at 24 hpi (Figure 4F).

### Depletion of STAT3 Blunts the Induction of ICD Markers in Melanoma Cells Upon NDV/FMW Infection

**Figure 5:**
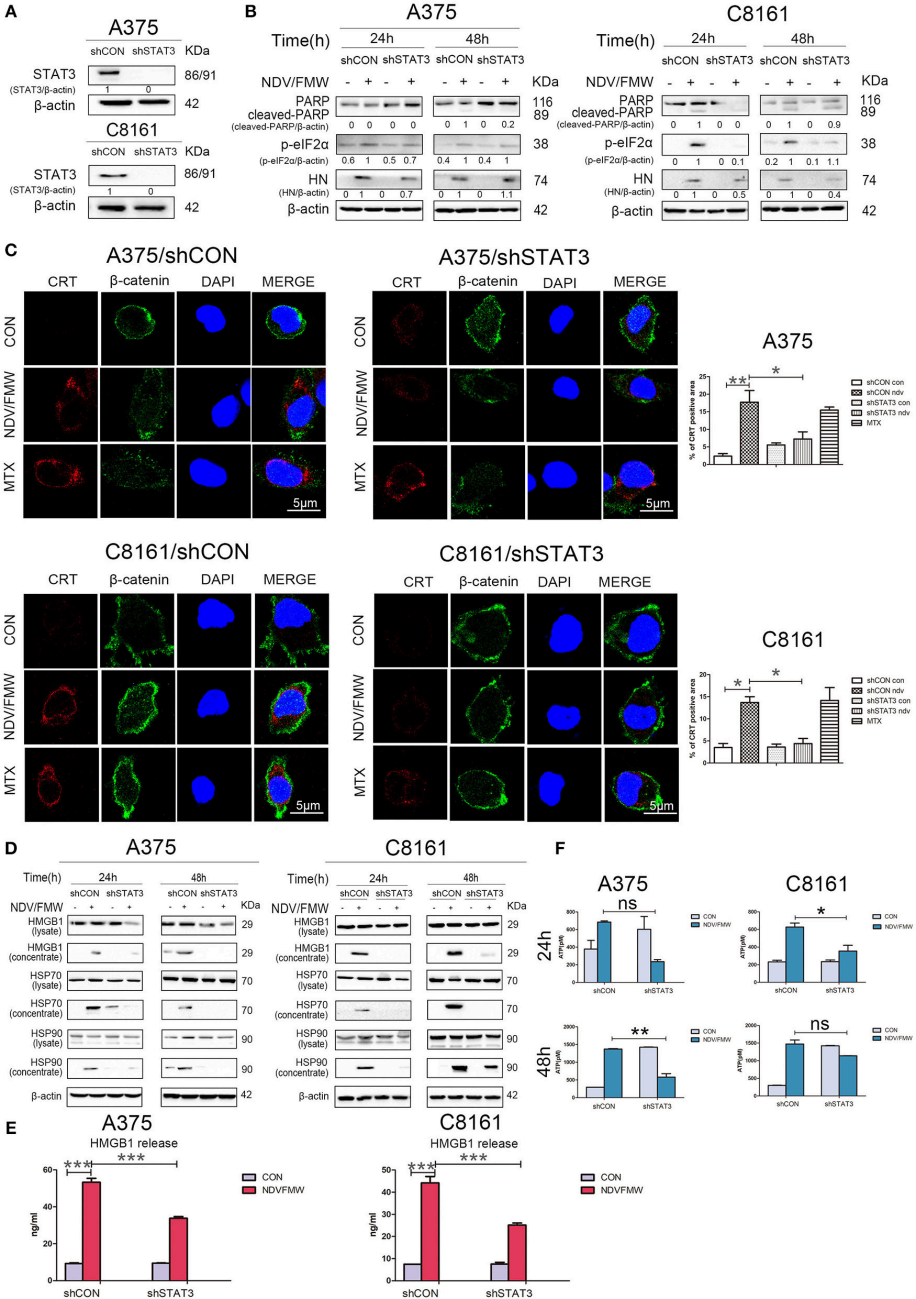
Effects of depletion of STAT3 on NDV/FMW-triggered immunogenic cell death. **(A)** A375 and C8161 cells with a stable knockdown of STAT3 (shSTAT3) and control cells (shCON) were tested by IB analysis. β-actin was used as a loading control. **(B–E)** STAT3-depleted cells and control cells were infected or mock-infected with NDV/FMW (MOI = 1). **(B)** After 24 and 48 h, the expression of cleaved-PARP, p-eIF2α and HN was determined by IB analysis. β-actin was used as a loading control. Data shown are representative of three independent experiments. **(C)** After 48 h, CRT exposure (red) was tested by confocal microscopy. MTX was used as a positive control. β-catenin was used as a membrane marker (green). DAPI was used for nuclear staining (blue). ImageJ software was used to calculate the percentage of CRT positive area (*p < 0.05, **p < 0.01). Images were obtained using confocal microscopy. Representative images are shown for three independent experiments. **(D)** After 24 and 48 h, the expression of HMGB1 and HSP70/90 in whole cell lysates and concentrated supernatants were measured by IB analysis. β-actin was used as a loading control. Data are representative of two independent experiments. **(E)**After 48 h, the release of HMGB1 in cell supernatants was detected by ELISA (***p < 0.001). Data shown are representative of three independent experiments. **(F)** After 24 and 48 h, extracellular ATP was measured by ELISA (*p < 0.05, **p < 0.01, n.s = not significant). Data are representative of three independent experiments.

To exclude the possible off-target effects by C188-9, we stably knockdown STAT3 with lentivirus-mediated shRNA targeting STAT3 in both A375 and C8161 cells. The knockdown efficiency was confirmed by immunoblot assay (Figure 5A). Consistent with the effects by C188-9 on NDV/FMW-mediated cell death in melanoma cells, STAT3 depletion also decreased NDV/FMW-triggered PARP cleavage and eIF2α phosphorylation (Figure 5B). Notably, the induction of ICD markers including CRT exposure, ATP secretion, and HMGB1 as well as HSP70/90 release upon NDV infection was severely attenuated in STAT3-depleted A375 cells compared with that in control shRNA-treated A375 cells (Figures 5C–F, left panels and Supplementary Figure 3A, upper panel). Similar results were obtained in C8161 cells (Figures 5C–F, right panels and Supplementary Figure 3A, lower panel).

### Supernatants Derived From NDV/FMW-infected Melanoma Cells Reduce Tumor Growth in Mice

**Figure 6:**
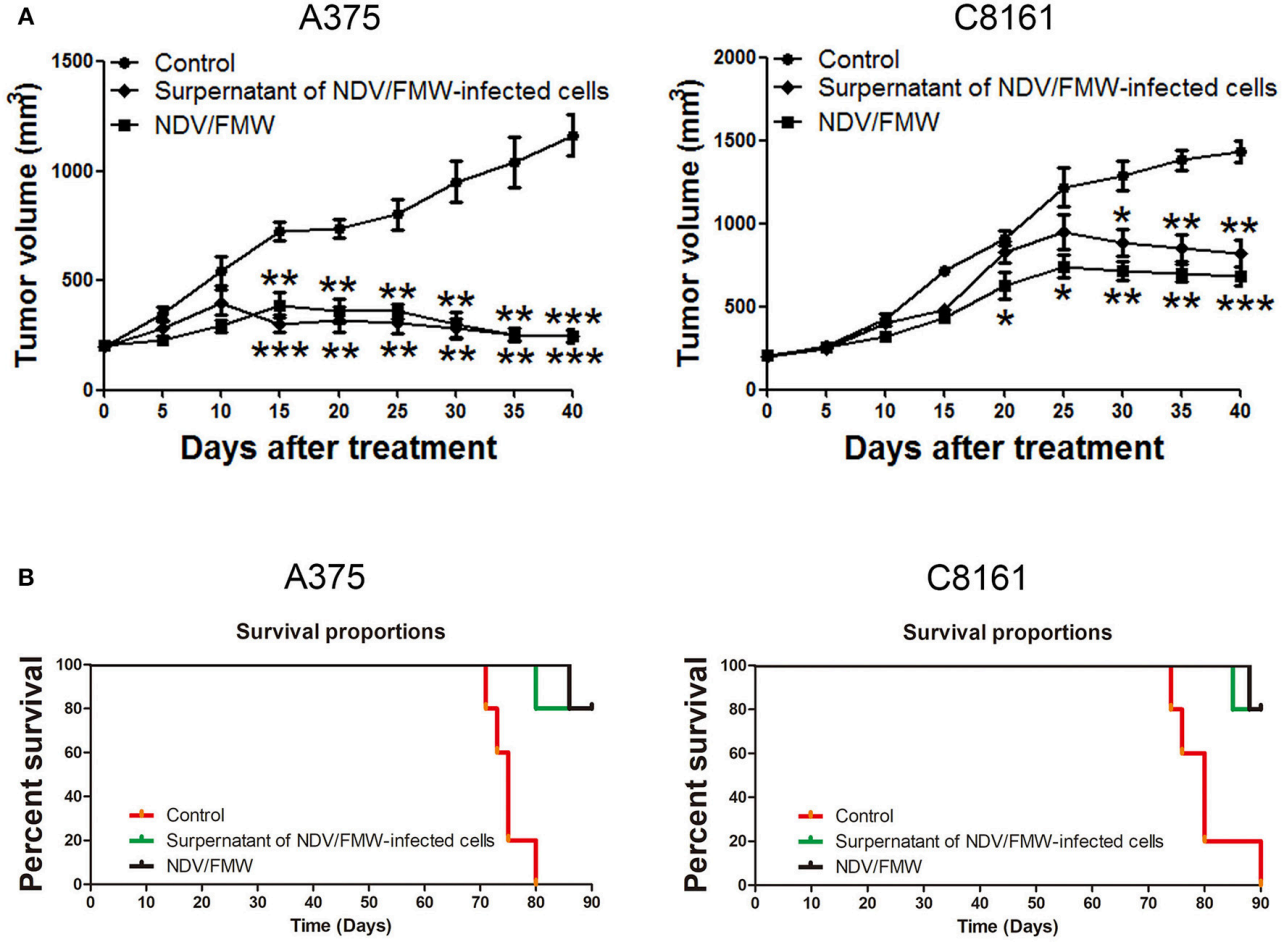
Effects of supernatants derived from NDV/FMW-infected melanoma cells on tumor growth in mice. **(A)** A375 and C8161 cells were subcutaneously inoculated into the flank. When tumors reached 100 mm^3^, tumor-bearing mice were intratumorally inoculated with either PBS, the concentrated cell-free supernatants of NDV/FMW (50 μl), or NDV/FMW every 3 days. Tumor volumes were measured at 5 day intervals for 40 days after injections and expressed as the Mean ± SD (*n* = 5) and represented as tumor volume-time curves. **(B)** Kaplan-Meier analysis of survival rates of each group was checked to last for 90 days from the day of tumor implant (*p < 0.05, **p < 0.01, ***p < 0.001).

We previously showed that the supernatants from NDV/FMW-infected lung cancer cells suppress tumor growth *in vivo* (45). To investigate whether the supernatants from NDV/FMW-infected melanoma cells could inhibit melanoma growth *in vivo*, the conditioned medium was collected, concentrated and irradiated with UV to inactivate the infectious virus. Mice bearing A375-derived tumors were intratumorally injected with NDV/FMW, the concentrated supernatants or vehicle. As shown in Figure 6A, the supernatants significantly reduced melanoma growth compared to vehicle-treated tumors. As expected, NDV/FMW injection significantly decreased tumor growth (Figure 6A). In addition, intratumoral administration of the concentrated supernatants evidently prolonged the survival of mice bearing A375-derived tumors compared to mice intratumorally injected with PBS (Figure 6B).

## Discussion

Our study declared and evidenced that oncolytic NDV/FMV provoked the expression and release of ICD imprints in melanoma cells. Besides this, we also validate the diverse fashion of cell death which further contributes to NDV/FMV-induced release of ICD markers to a major extent. Of note, the transcription factor STAT3 plays a critical role in the induction of ICD in melanoma cells exposed to NDV/FMW.

It has been shown that multiple modes of cell death such as apoptosis, are involved in either natural or recombinant oncolytic NDV-mediated cytolytic activities in melanoma (64–68). However, whether oncolytic NDV elicits ICD in melanoma has not been investigated. In the current investigation, we found that oncolytic NDV/FMW elicits the induction of several known ICD markers, such as CRT exposure, ATP secretion, and HMGB1 as well as HSP70/90 release in melanoma cells. Furthermore, the anti-melanoma effects by oncolytic NDV-induced ICD were attested in a xenograft model. Therefore, our data strongly suggest that oncolytic NDV can be a potent ICD inducer in melanoma cells. This observation is generally in line with our previous work in lung cancer cells and others in glioma (44, 45), indicating that oncolytic NDV could elicit ICD in diverse types of cancers. In addition, we observed that pharmacological inhibition of apoptosis, autophagy, necroptosis and ER stress suppresses NDV/FMW-induced release of several ICD markers in melanoma cells, suggesting that diverse forms of cell death might contribute to the induction of ICD in melanoma cells upon oncolytic NDV infection. Of interest, work by Koks et al. indicated that necroptosis but not caspase signaling contributes to oncolytic NDV-triggered ICD in glioma while our previous work in lung cancer cells showed that the induction of ICD by oncolytic NDV relies on autophagy other than apoptosis or necroptosis (44, 45). These differences may reflect some complexity in the regulation of the induction of ICD by NDV in cancers. Therefore, to fine modulate NDV-induced ICD, these differences should be taken into consideration for the combination usage of NDV with other agents.

The transcription factor STAT3 is often hyperactivated in melanoma and is a potential target for melanoma therapy (59, 63, 69, 70). One of the important findings of the present study is that targeting STAT3 by either pharmacological inhibition with STAT3 inhibitor C188-9 or shRNA-mediated depletion suppresses oncolytic NDV-primed expression and release of ICD markers in melanoma cells. It should be pointed out that our data are somewhat not consistent to a recent study showing that deletion of STAT3 stimulates one of the hallmarks of ICD, namely the production of type 1 interferons, but not other ICD markers in fibrosarcoma cells (58, 60). Nevertheless, these studies reinforce that targeting STAT3 as a potential strategy for modulating anticancer immunotherapy. Given that targeting STAT3 by the STAT3 inhibitors such as C188-9 has been evaluated in early phase clinical trials for advanced-stage cancers (NCT03195699) (61–63), it should be taken into caution for the rational design of combinatorial approaches using chemotherapy to boost oncolytic NDV-induced ICD.

Of great importance, oncolytic NDV has been demonstrated to elicit an antitumor immune response in melanoma (37, 39, 67, 71, 72). Furthermore, recent studies revealed that oncolytic NDV-based virotherapy overcomes systemic tumor resistance to immune checkpoint blockade in melanoma (42, 43), underscoring the potential role of oncolytic NDV in cancer immunotherapy. In this regard, our data highlight the importance of understanding the mechanism(s) underlying the antitumor immune response induced by oncolytic NDV.

## Ethics Statement

Animal experiments were conducted at Dalian Medical University (Dalian, China), in compliance with the national guidelines for the care and use of laboratory animals. All animal experiments were approved by the experimental animal ethics committee of Dalian Medical University.

## Author Contributions

XS, XW, XG, GZ, SM, and QX thought of the study, designed the experiments, performed the experiments, analyzed the data, and wrote the paper. KJ, TY, JC, JF, LG, and SW performed the experiments. All authors read and approved the final manuscript.

### Conflict of Interest Statement

The authors declare that the research was conducted in the absence of any commercial or financial relationships that could be construed as a potential conflict of interest.
