# Supplementary material for: STAT3 Contributes To Oncolytic Newcastle Disease Virus-Induced Immunogenic Cell Death in Melanoma Cells
Source: Front Oncol. 2019 May 29;9:436. doi: 10.3389/fonc.2019.00436 (PMC6548873; doi:10.3389/fonc.2019.00436)
Supplement: Supplementary file 1 [file Data_Sheet_1.PDF]

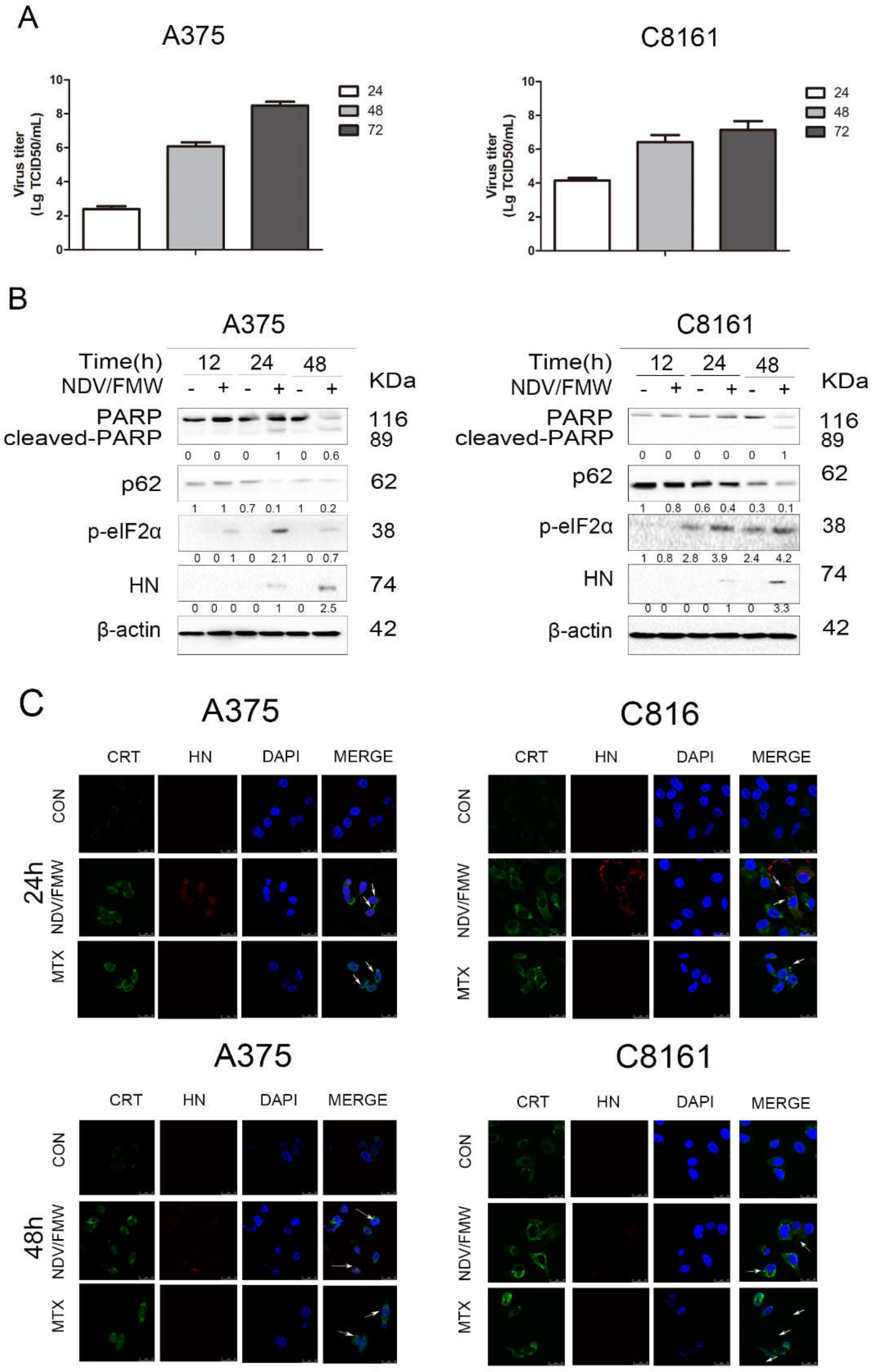

Supplementary Figure 1. NDV/FMW induces cell death in melanoma cells and CRT exposure.

(A) A375 and C8161 cells were infected with NDV/FMW at a multiplicity of infection (MOI) of 0.01. Virus yield in NDV/FMW infected cells was examined by multi-step viral growth curves. Data are presented as the mean ± SD for triplicate assays. (B) A375 and C8161 cells were infected with or without NDV/FMW (1 MOI) for 12, 24 and 48 h, the expression of cleaved poly (ADP-ribose) polymerase (PARP),

p62, p-eIF2α and hemagglutinin-neuraminidase protein (HN) were examined by immunoblot analysis (n = 2). β-actin was used as a loading control. The relative quantity of protein was assessed by Image Lab software. Immunoblots shown are representative of two independent experiments. (C) A375 and C8161 cells were infected as the same in (B), the cells were stained with an anti-CRT antibody (green) and anti-HN antibody (red), and assessed by confocal imaging at 24 and 48 hpi of NDV/FMW (MOI=1). DAPI was used for nuclear staining (blue). Mitoxantrine (MTX) was used as a positive control. Images are representative of three independent experiments.

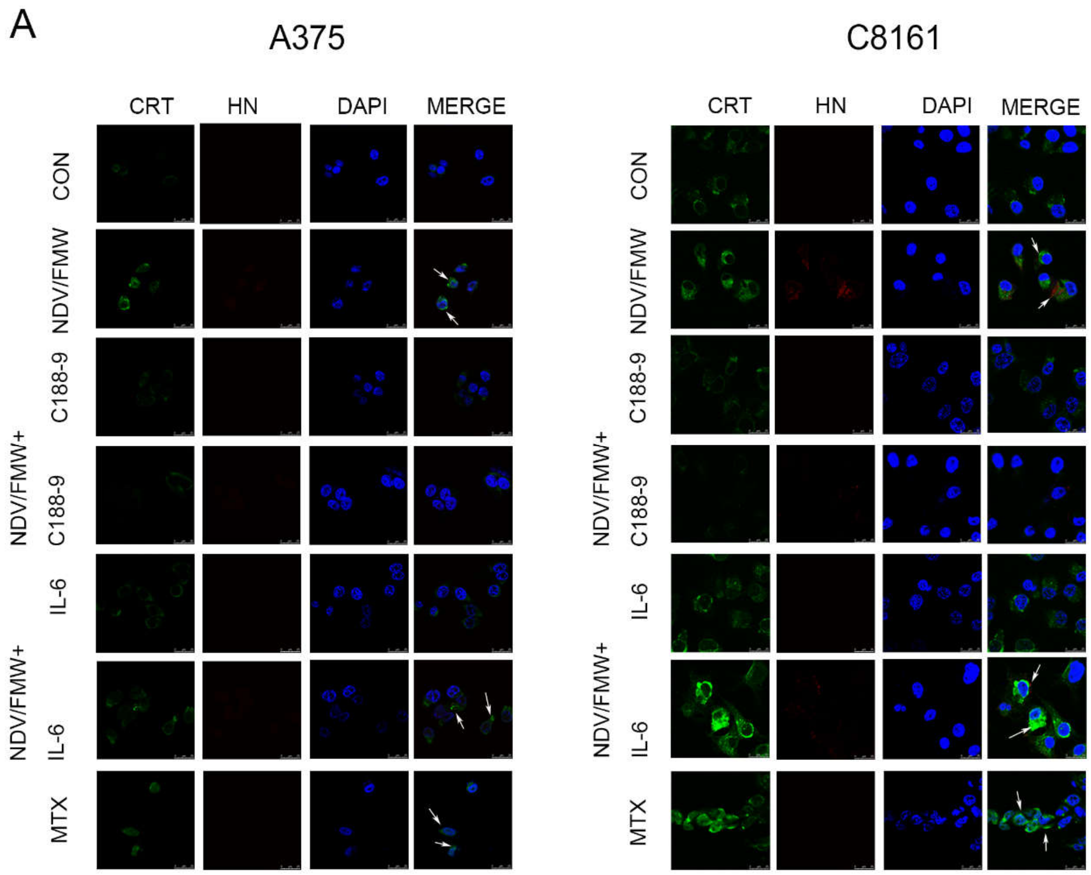

Supplementary Figure 2. STAT3 inhibition exerts effects on the exposure of CRT induced by NDV/FMW.

(A) A375 and C8161 cells were pre-treated with C188-9 (0.9 μM) and IL-6 (30 ng/mL) for 1 h, and then the cells were infected or mock-infected with NDV/FMW (MOI=1) for 48 h. the cells were stained with an anti-CRT antibody (green) and anti-HN antibody (red) and assessed by confocal imaging. DAPI was used for nuclear staining (blue). MTX was used as a positive control. Images are representative of three independent experiments.

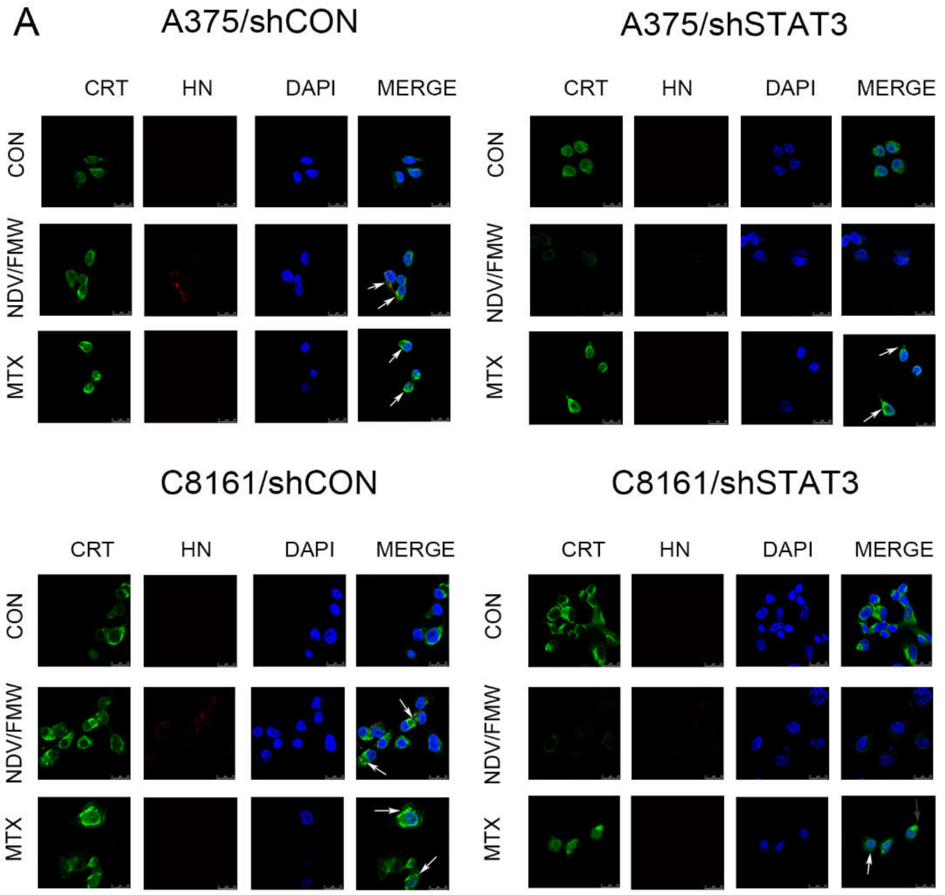

Supplementary Figure 3. Effects of depletion of STAT3 on NDV/FMW-triggered CRT exposure.

(A) STAT3-depleted cells (shSTAT3) and control cells (shCON) were infected or mock-infected with NDV/FMW (MOI=1). After 48h, the cells were stained with an anti-CRT antibody (green) and anti-HN antibody (red) and assessed by confocal imaging. DAPI was used for nuclear staining (blue). MTX was used as a positive control. Images are representative of three independent experiments.
